# Exploring the Relationship Between Sleep Disturbances, Gut Microbiota, and Cognition in Patients with Heart Failure: A Cross-Sectional Study

**DOI:** 10.3390/medicina62091643

**Published:** 2026-08-27

**Authors:** Hesam A. Varpaei, Stuart F. Quan, Lorraine B. Robbins, Fabrice I. Mowbray, Mathew Reeves, Milind Karve, Lei Gao, Pallav Deka

**Affiliations:** 1 Perioperative Sleep and Brain Health Laboratory, Department of Anesthesia, Critical Care & Pain Medicine, Massachusetts General Hospital, Harvard Medical School, Boston, MA 02114, USA; hvarpaei@mgh.harvard.edu (H.A.V.); lgao@mgh.harvard.edu (L.G.); 2 Division of Sleep and Circadian Medicine, Mass General Brigham, Harvard Medical School, Boston, MA 02115, USA; 3 College of Nursing, Michigan State University, East Lansing, MI 48824, USA; robbin76@msu.edu (L.B.R.); mowbray3@msu.edu (F.I.M.); 4 Department of Epidemiology and Biostatistics, College of Human Medicine, Michigan State University, East Lansing, MI 48824, USA; reevesm@msu.edu; 5 Capital Cardiology, Sparrow Hospital, University of Michigan Health, Lansing, MI 48912, USA; karavem@msu.edu; 6 College of Nursing, Wayne State University, Detroit, MI 48202, USA

**Keywords:** heart failure, sleep, microbiome, cognition, short-chain fatty acids

## Abstract

*Background and Objectives*: Poor sleep quality and gut microbiota dysbiosis are independently associated with cognitive decline, yet their effects on cognition in heart failure (HF) patients remain unexamined. To assess the independent associations of sleep quality and gut microbiota diversity with cognition in patients with HF. *Methods*: This exploratory/pilot cross-sectional study enrolled adults with chronic stable HF from an outpatient cardiology clinic. Participants completed the Pittsburgh Sleep Quality Index (PSQI) and Montreal Cognitive Assessment (MoCA). Fecal samples were analyzed for gut microbiota composition, including Shannon diversity and Simpson indices. Multivariable linear regression analysis of MoCA scores with sleep quality and alpha diversity (Shannon index) as primary exposures, adjusted for age, sex, and left ventricular ejection fraction (LVEF) was conducted. *Results*: Of 59 enrolled patients (mean age 73.3 ± 10.8 years; 72.7% male; 86.4% White), 40 provided fecal samples. Analysis of microbiota data revealed a composition dominated by *Bacillota* (63.7%). In multivariable regressions, poor sleep quality was independently associated with lower MoCA scores (β = −3.01 to −3.88, *p* < 0.01). Similarly, Shannon diversity was also associated with lower MoCA (β = −3.45, *p* < 0.05). Beta-diversity analyses showed no consistent association between overall microbial community composition and cognition. No significant interactions between microbiota diversity and sleep variables were identified (all *p* > 0.20). *Conclusions*: Sleep quality and gut microbiota alpha diversity are independently associated with cognitive function in HF. Microbial pathways favoring short-chain fatty acid production align with better sleep, while lipopolysaccharide-related pathways associate with poorer sleep and cognition.

## 1. Introduction

Heart failure (HF) affects millions, and its treatment costs are projected to reach $69 billion by 2030, driven by high readmission rates of 18–23% at 30 days and 30–34% at 90 days [1,2,3]. Cognitive impairment (CI) compounds this burden: it is highly prevalent in HF [4,5,6], impairs self-care and medication adherence [7,8,9], and independently increases mortality risk [10,11]. HF itself appears to drive CI through reduced cerebral perfusion, systemic inflammation, and neurohormonal dysregulation [12,13], though the available evidence remains observational [11,12,13]. Identifying modifiable contributors to CI in HF is therefore an urgent clinical priority.

Poor sleep quality and gut microbiota dysbiosis are two potentially modifiable factors. Sleep disturbances affect 15–22% of the general population [14] but are far more prevalent in HF, ranging from 52.9% to 96% [15,16]. Poor sleep is also linked to worse cognition in older adults [17]. Gut microbiota dysbiosis in HF is characterized by reduced alpha diversity, depletion of short-chain fatty acid (SCFA)-producing genera (*Lachnospiraceae*, *Ruminococcaceae*), and expansion of pathobionts (Escherichia, Streptococcus), promoting systemic inflammation through lipopolysaccharide translocation, a process worsened by HF-associated intestinal hypoperfusion [18,19]. Critically, SCFA-producing bacteria modulate cognition and sleep via the gut–brain axis through vagal signaling, blood–brain barrier regulation, and neuroinflammation. HF patients show significantly lower alpha diversity than healthy adults [20,21], and dysbiosis associates with both sleep disruption [22,23,24,25] and CI in non-HF populations [26,27]. Microbiome-modulating interventions have improved sleep [28,29,30] and cognition [28,29,31] in non-HF samples, but evidence in HF is absent.

A few prior HF studies have examined sleep quality [15,16,32] or microbiome features in isolation [18,19], or relied on secondary sleep datasets without microbiome characterization [33,34]. The independent and joint contributions of sleep quality and gut microbiota to CI in HF remain unexamined. The primary aim of this study was to assess the independent associations and interaction effects of sleep quality and gut microbiota (including alpha diversity and species abundance) with cognition in patients with HF adjusted for age, sex, and left ventricular ejection fraction (LVEF). We also explored sleep quality, sleep duration, and sleep efficiency as potential effect modifiers of the association between gut microbiota and cognitive function. We hypothesized that both poor sleep quality and reduced gut microbiota diversity would be independently associated with lower cognitive performance, and that the association between sleep quality and cognition would be stronger in patients with greater gut microbiota dysbiosis.

## 2. Materials and Methods

This pilot/exploratory cross-sectional study used a convenience sampling method with participants completing all measurements at a single time point. This study was approved by the Institutional Review Board of Michigan State University, Biomedical and Health Institutional Review Board (ID: STUDY00011084, approval date: 25 September 2024), and participants provided written informed consent prior to enrollment.

### 2.1. Subjects and Setting

The target population of this study was adults with chronic stable HF (defined as no hospitalization, symptom change, or medication adjustment in the preceding month). Inclusion criteria were as follows: adults > 18 years of age; diagnosed with HF (I–IV NYHA Classification; including both HF with reduced ejection fraction and HF with preserved ejection fraction) based on medical record review and clinician report; and able to speak and read English. Sleep disorders and sleep medication use were not used as inclusion or exclusion criteria to preserve clinical representativeness; these variables were assessed as study measures. We excluded adults with clinically diagnosed dementia (e.g., Alzheimer’s disease) or clinically diagnosed Crohn’s disease and ulcerative colitis (as they have very different gut microbiota than groups without these health conditions [28,35]), night shift workers, patients with hospitalization in the previous month, or report of taking antibiotics within the past 30 days.

### 2.2. Patients’ Recruitment and Data Collection

Participants were recruited using a convenience sample from an outpatient cardiology clinic during their regular visit to see the provider. After obtaining informed consent, the first author (trained and certified in the Montreal Cognitive Assessment [MoCA]) conducted cognition tests (using MoCA), then provided participants with paper surveys for completion (demographics, Pittsburgh Sleep Quality Index [PSQI]; Table 1). After completion of all surveys, a home fecal sample collection kit and a prepaid postbox for mailing the fecal sample back were provided to each participant. Participants were compensated for their time and effort with a $20 gift card for completing the survey and an additional $20 for providing a fecal sample.

### 2.3. Exposures

The two primary exposures in this study were global sleep quality [36] and alpha diversity index (Shannon Index, Chao1, Simpson; all continuous). The PSQI consists of 19 items (global score range = 0 to 21) and assesses overall sleep quality, where poor sleep quality is defined as PSQI > 5 [36]. The PSQI also provides various other sleep quality metrics, including daytime sleepiness, total sleep time (TST; cutoff > 7 h), sleep latency (SL; cutoff > 20 min), and sleep efficiency (SE; cutoff < 85%).

### 2.4. Outcomes

Cognition was assessed continuously using the Montreal Cognitive Assessment (MoCA), a 30-point screening measure of global cognitive performance encompassing attention, memory, language, visuospatial abilities, executive function, and orientation [37]. The MoCA is a valid cognitive screening instrument used in older adults with HF, with reported sensitivity and specificity of 82% and 91%, respectively, for identifying possible mild cognitive impairment [38]. The standard education correction of one additional point was applied for participants with 12 years of education or less. For descriptive purposes only, MoCA scores <26 were classified as screening-positive for possible cognitive impairment and were not interpreted as a clinical diagnosis [39,40].

### 2.5. Gut Microbiota Analysis

Gut microbiota composition was characterized using 16S rRNA gene sequencing. Raw paired-end sequencing reads were processed in R using the DADA2 pipeline (Version 1.34.0). Reads were quality-filtered and trimmed based on quality profiles, and sequencing errors were modeled to infer exact amplicon sequence variants (ASVs) without clustering. Forward and reverse reads were merged, and chimeric sequences were identified and removed. Taxonomic assignment was performed using the SILVA reference database (release 138.2, April 2023). QIIME2 (Version 2024.10.1) was used for additional downstream processing and data handling where appropriate. An ASV abundance table was generated for subsequent analyses.

Four alpha diversity metrics of interest were calculated from the ASV count table using the vegan package in R. The Shannon Diversity Index was used as the primary exposure measure of within-sample microbial diversity, reflecting both richness and evenness. Additional indices (Simpson, Chao1, and Observed ASVs) were calculated for descriptive and correlation analysis. Phyloseq (R 4.5.2 package) was used to structure and manage microbiome data objects for analysis. All alpha diversity measures were treated as continuous variables in correlation and regression models. Definitions of alpha diversity metrics are below:

- Shannon Diversity Index: The Shannon index quantifies both species richness and evenness within a community by incorporating the proportional abundance of each taxon. It increases as the number of taxa and the evenness of their distribution increase [41].
- Simpson’s dominance index ($D=\sum p_{i}^{2}$) measures community concentration, with higher values indicating greater dominance and lower alpha diversity [42].
- Chao1 is a nonparametric estimator of species richness that accounts for undetected taxa by incorporating the number of singletons and doubletons in the dataset [43].
- Observed richness represents the raw count of distinct taxa (ASVs) detected in a sample. It reflects species richness only and does not account for abundance distribution or unseen taxa [44].

Beta diversity was quantified using Bray–Curtis dissimilarity calculated from relative-abundance profiles and visualized using principal coordinates analysis. Principal Coordinates Analysis (PCoA) Axis 1 and Axis 2 explained 13.84% and 9.35% of the variation, respectively. Because Bray–Curtis is nonphylogenetic, no phylogenetic tree was used. Overall associations between microbial community composition and MoCA were evaluated using Permutational Multivariate Analysis of Variance (PERMANOVA). Separate exploratory linear regression models examined associations of MoCA with the first two PCoA axes while adjusting for sleep quality, age, sex, and LVEF (Table 2).

Taxonomic abundance tables were aggregated at the phylum and genus levels. For each sample, raw sequence counts were normalized to relative abundance by dividing taxon-specific counts by the total reads per sample to account for differences in sequencing depth. Mean relative abundance for each taxon was then calculated across all participants. Genera present in fewer than 20% of samples were excluded to reduce sparsity and improve stability of downstream analyses. Taxa were subsequently ranked based on mean relative abundance to identify the most predominant phyla and genera within the cohort.

The Analysis of Compositions of Microbiomes (ANCOM; Tables S6–S10) was used to determine differences in the microbial community among microbiomes. The function ANCOM-BC estimates the unknown sampling fractions, corrects the bias induced by their differences through a log-linear regression model, and identifies taxa that are differentially abundant according to the variable of interest [45]. ANCON-BC tests were carried out for all taxonomic levels (Phylum, Class, Order, Family, Genus, and Species). The criteria for differential abundance analysis include removing zero-sum taxa (>0), zero-variance taxa, very low-depth samples (>1000), and a minimum proportion of samples (>0.05).

Microbial functional potential was inferred from 16S rRNA ASV profiles using PICRUSt2 [Picrust2-v2.6.3] and the PICRUSt2-SC reference database based on GTDB release 214. Prediction quality was assessed using the Nearest Sequenced Taxon Index (NSTI), with ASVs exceeding the PICRUSt2 default threshold (NSTI > 2) excluded before functional prediction (Table S1). ASVs were placed on a reference phylogenetic tree and predicted KEGG orthologue (KO) abundances were generated through ancestral-state reconstruction and aggregated at the sample level. Prior to differential abundance analysis, predicted KOs were filtered to retain those detected in ≥10% of samples, consistent with the ANCOM-BC2 prevalence threshold. This filtering retained 6363 of 6818 predicted KOs (93.3%). On the other hand, no minimum library-size cutoff was applied, because PICRUSt2-predicted KO abundances represent inferred functional profiles rather than raw sequencing depths. Sleep efficiency (SE) was entered into the ANCOM-BC2 models as a continuous proportion (0–1; observed range 0.56–1.00; mean 0.83 ± 0.15 SD), rather than as a percentage (0–100) or a dichotomized variable; reported log-fold changes (LFC) therefore reflect change per 1.0-unit increase in SE and were additionally expressed per 0.10-unit (10-percentage-point) increase for interpretability (Figures S4 and S5). To assess the reliability of individual LFC estimates, each KO was evaluated for overall sparsity (percentage of samples with zero predicted abundance) and for the minimum number of non-zero observations on either side of the median SE split, identifying potential quasi-complete separation and inflated estimates for sparse features (Table S2). Associations between each KO and outcomes were evaluated using ANCOM-BC log-linear models without covariate adjustment. ANCOM-BC corrected for sampling-fraction bias, and resulting *p*-values were adjusted for multiple testing using the false discovery rate. Significant KOs were grouped post hoc into predefined functional pathways, and complete KO-level results are provided in Supplementary File S2.

### 2.6. Statistical Analysis

All analyses were conducted using R (RStudio). Continuous variables are presented as mean ± standard deviation and categorical variables as frequency and percentage. Pearson correlation coefficients were calculated for continuous variables. Correlation matrices were generated to visualize pairwise relationships between sleep measures (TST, SL, SE, and PSQI global score), alpha diversity indices (e.g., Shannon, Simpson, Chao1), demographic and clinical characteristics, and MoCA scores.

Multiple linear regression models were used to examine associations of sleep and gut microbiota diversity with MoCA scores. Sleep exposures were dichotomized as poor sleep quality (PSQI > 5), low sleep efficiency (<85%), and short sleep duration (<7 h). Beta diversity was quantified using Bray–Curtis dissimilarity, and overall associations between microbial community composition and modified MoCA scores were evaluated using PERMANOVA across taxonomic levels. Principal coordinates analysis was used for visualization, and the first two PCoA axes were included in secondary exploratory regression models with one sleep exposure, age, sex, and LVEF. Separate alpha-diversity models included one sleep exposure, one alpha-diversity metric (Shannon, Chao1, or Simpson), age, sex, and LVEF. Interaction models additionally included the corresponding sleep-by-alpha-diversity interaction term. Linearity of continuous predictors was assessed using component-plus-residual plots and the Rainbow test. Given the limited sample size, all analyses were considered exploratory and hypothesis-generating. Species-level analyses were conducted separately for the three most abundant taxa identified based on mean relative abundance across participants. Significant Kyoto Encyclopedia of Genes and Genomes (KEGG [46,47]) orthologs (KO) were grouped post hoc into predefined functional pathways (short-chain fatty acids (SCFAs), trimethylamine N-oxide (TMAO) precursors, and lipopolysaccharides) based on established annotations to enable biologically interpretable synthesis of results. Effects are reported as log-fold change (LFC), representing the logarithmic difference in relative abundance per unit increase in the predictor, where positive values indicate higher abundance and negative values indicate lower abundance. Statistical significance was defined as *p* < 0.05 for all results except KEGG-KO, for which statistical significance was determined using FDR-adjusted q-values, with q < 0.05 considered statistically significant (Benjamini–Hochberg false discovery rate [48] correction applied to control for multiple testing).

## 3. Results

### 3.1. Participant Characteristics

**Figure 1:**
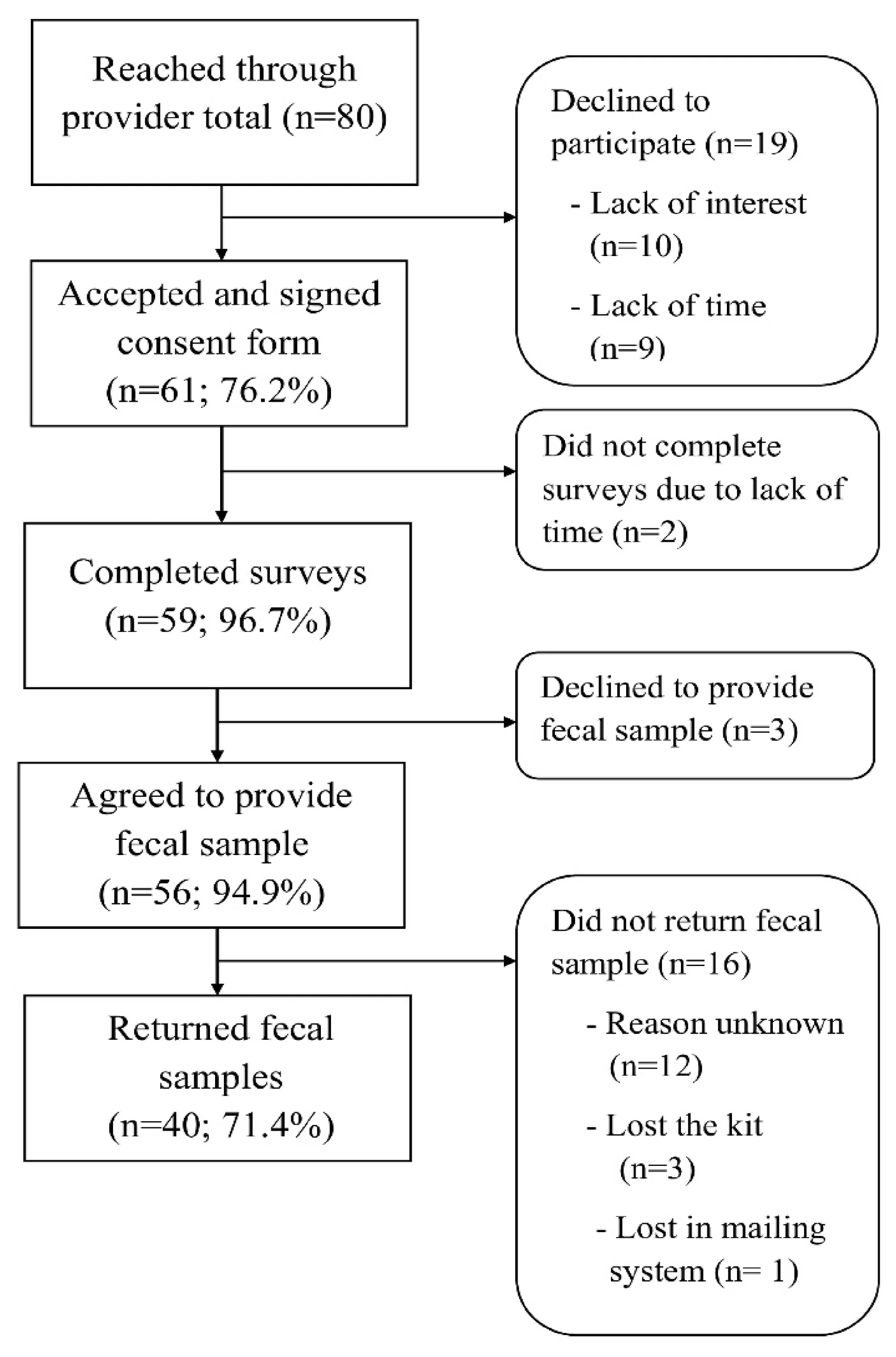
Flow diagram of study recruitment.

A total of 59 patients (n = 43 [72.8%] male; Figure 1) were enrolled and completed surveys (White/Caucasian n = 51 [86.4%]; mean age: 73.29 ± 10.80 [8.4% were <aged 65]). Three participants declined to provide fecal samples. Over two-thirds of the sample (n = 40; 71.4%) mailed back fecal samples. Hypertension (n = 58; 98%) and hyperlipidemia (n = 57; 97%) were the most prevalent comorbidities. On average, fecal samples were received 2 weeks after recruitment (Table 1). Approximately one third of the included samples had heart failure with preserved ejection fraction (mean LVEF = 54.1 ± 8.43%), and the rest heart failure with reduced ejection fraction (mean LVEF = 38.9 ± 7.11%).

**Figure 2:**
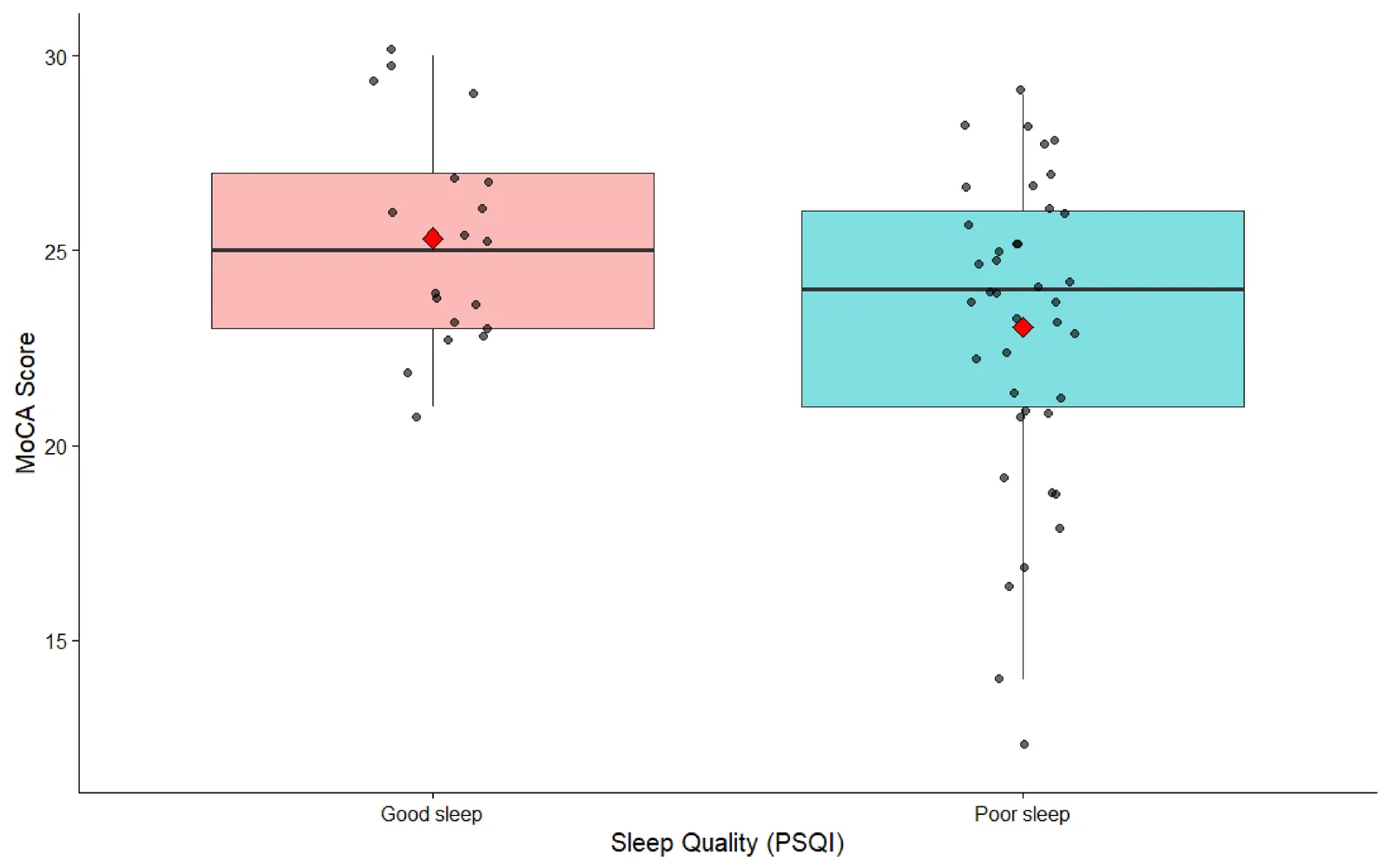
Boxplot comparing the MoCA scores of good quality sleep (PSQI ≤ 5) and poor quality sleep (PSQI > 5) among patients with HF.

The majority of participants screened as having possible cognitive impairment, with 40 (67.8%) scoring below 26 on the MoCA. Poor sleep quality (PSQI > 5; Figure 2) was associated with a higher proportion of CI compared to good sleepers (72.5% vs. 57.9%), as was adequate sleep duration, where those meeting sleep duration criteria paradoxically showed higher CI rates than short sleepers (75.8% vs. 57.7%). Sleep efficiency showed minimal separation between groups, with CI rates of 69.0% in those with good efficiency and 66.7% in those with poor efficiency.

### 3.2. Microbiota Diversity

Participants with CI (MoCA < 26; Tables S1 and S2) demonstrated significantly higher alpha diversity compared to those with normal cognition across multiple metrics, including Shannon diversity (3.70 ± 0.32 vs. 3.38 ± 0.44, *p* = 0.008), Chao1 richness (262.3 ± 66.3 vs. 215.6 ± 76.9, *p* = 0.048), and Simpson index (0.050 ± 0.014 vs. 0.070 ± 0.032, *p* = 0.012), with the paradoxical direction suggesting higher diversity in the cognitively impaired group. In contrast, no significant differences in alpha diversity were observed across PSQI sleep quality categories (all *p* > 0.52), sleep efficiency groups (all *p* > 0.32), or sleep duration groups (all *p* > 0.08), though adequate sleepers showed a trend toward higher Chao1 richness compared to short sleepers (262.8 ± 82.3 vs. 223.2 ± 51.5, *p* = 0.075).

### 3.3. Microbiota Composition of the Sample

At the phylum level (Figure S1), the gut microbiota of the cohort was dominated by *Bacillota* (mean relative abundance 64%), followed by *Bacteroidota* (27%) and *Actinomycetota* (6%). Together, these three phyla accounted for more than 95% of the total microbial composition. Other phyla, including *Pseudomonadota* and *Fusobacteriota*, were present at substantially lower relative abundances (<4%). At the Genus level (Figure S2), the most abundant taxa included *Lachnospiraceae* FCS020 group, *GCA-900066755*, *Faecalibacterium*, *(Clostridium) innocuum group*, and Porphyromonas.

### 3.4. Beta Diversity, Sleep, and Cognition Analysis

**Figure 3:**
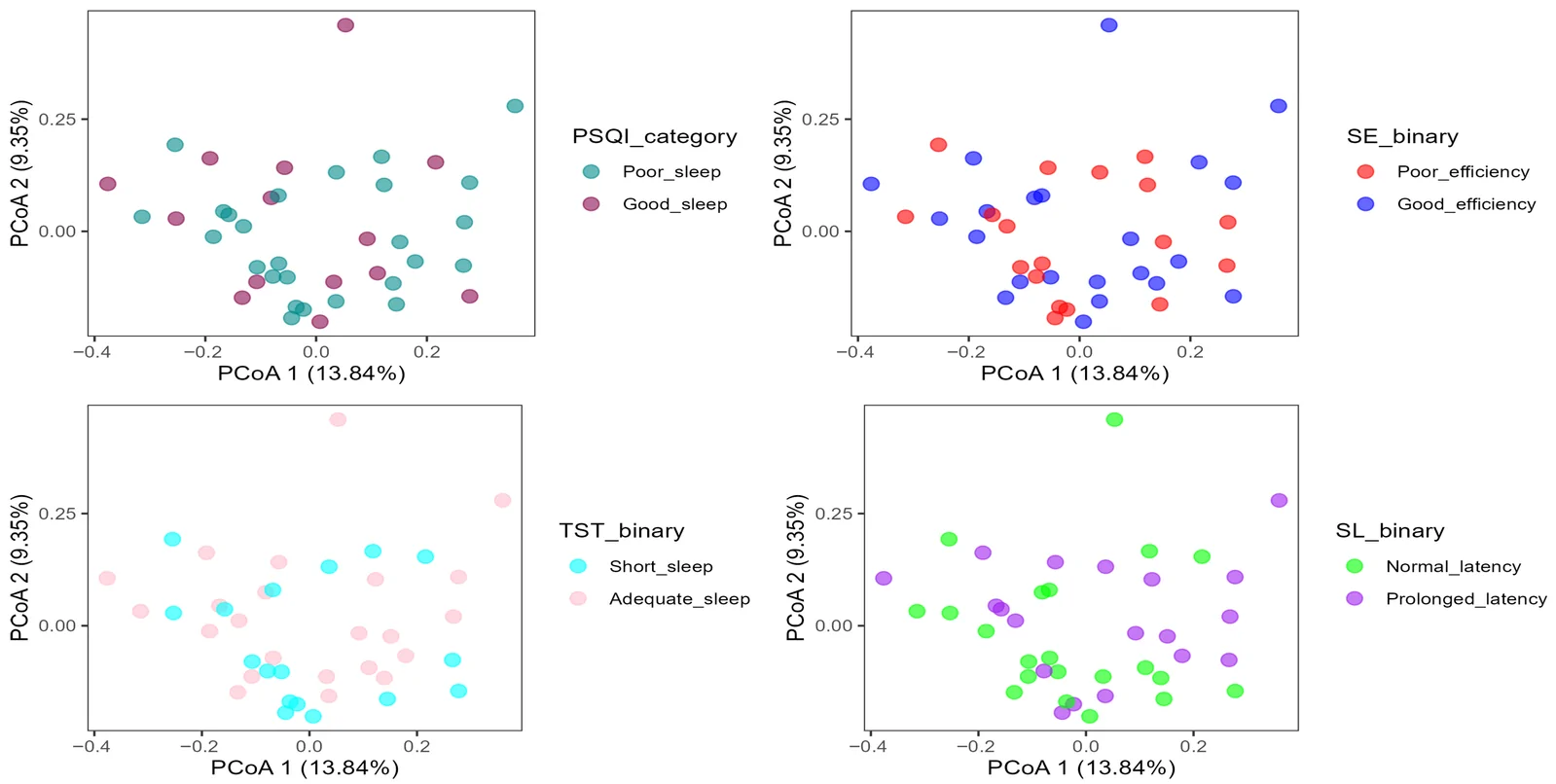
Principal coordinates analysis of Bray–Curtis dissimilarity according to sleep categories. Points represent individual participants, and colors indicate category membership. Poor sleep quality was defined as PSQI > 5; poor sleep efficiency as <85%; short sleep duration as <7 h; and prolonged sleep latency as >20 min. PCoA Axis 1 and Axis 2 explained 13.84% and 9.35% of the variation, respectively.

PCoA plots demonstrated substantial overlap between sleep-category groups, with no clear visual separation according to sleep quality, sleep efficiency, total sleep time, or sleep latency (Figure 3). PERMANOVA showed a nominal association between continuous MoCA scores and species-level microbial composition ($R^{2}=0.043$, p=0.033), whereas associations at the phylum, class, order, family, and genus levels were not statistically significant (Table 3). In the exploratory adjusted linear regression model (Table 2), poor sleep quality was associated with a 3.76-point lower MoCA score compared with good sleep quality (β=−3.76, p=0.006). Neither PCoA Axis 1 nor Axis 2 was associated with MoCA (p=0.332 and p=0.600, respectively). Older age was associated with lower MoCA scores (β=−0.23, p<0.001), whereas sex and LVEF were not significant. The model explained 46% of the variance in MoCA scores ($R^{2}=0.46$, overall p=0.001).

**Table 3:** PERMANOVA associations between modified MoCA score and Bray–Curtis beta diversity.

Taxonomic Level	df	Sum of Squares	*R* ^2^	F	*p* Value
Phylum	1	0.095	0.073	2.972	0.065
Class	1	0.102	0.061	2.448	0.072
Order	1	0.117	0.048	1.897	0.097
Family	1	0.134	0.038	1.488	0.152
Genus	1	0.240	0.038	1.507	0.094
Species	1	0.325	0.043	1.700	0.033

***Note.** PERMANOVA was conducted using Bray–Curtis dissimilarity matrices. R*^2^ *represents the proportion of variation in microbial community composition explained by the MoCA score.*

### 3.5. Alpha Diversity, Sleep, and Cognition Analysis

In multiple linear regression models adjusted for age, sex, LVEF, and microbiota alpha diversity, poor sleep quality was consistently associated with lower MoCA scores across Shannon, Chao1, and Simpson models, β = −3.22 to −3.88, *p* < 0.01 (Table 4). In contrast, short sleep duration and poor sleep efficiency were not significantly associated with MoCA scores in any model. Microbiota diversity indices showed mixed associations with cognition: Shannon diversity was negatively associated with MoCA across models, β = −3.45 to −4.45, *p* < 0.05 to 0.01, whereas Simpson diversity was positively associated with MoCA, β = 57.43 to 72.71, *p* < 0.05 to 0.01. Chao1 richness was not significantly associated with MoCA across any model. Overall, these findings suggest that global sleep quality, rather than sleep duration or sleep efficiency alone, was the sleep-related factor most consistently associated with cognitive performance, while the direction and significance of microbiota associations varied by alpha diversity index.

We tested for moderation of the gut microbiota-cognition relationship by sleep quality, duration, and efficiency. No significant interactions were identified (all *p* > 0.20), suggesting the associations between gut microbiota diversity and cognitive function operate independently of sleep quality in this sample (Table S5).

### 3.6. KEGG Orthologs Identified Functional Metagenomic

Across all bacterial ASVs used for functional prediction, NSTI values were generally low (mean = 0.096 ± 0.096; median = 0.085, IQR: 0.029–0.134). More than 91% of ASVs had NSTI ≤ 0.2, and none exceeded the default threshold of 2.0 (Table S1 and Figure S3), indicating strong representation of the detected taxa among sequenced reference genomes and supporting the robustness of PICRUSt2 functional predictions.

Metabolic pathways associated (Table 5) with the production of SCFAs, including propionate kinase (LFCSE = 9.92), propionyl-CoA: succinyl-CoA transferase (LFCSE = 10.82), and malate-CoA ligase (LFCSE = 16.04), were consistently positively associated with improved sleep duration and efficiency. Similarly, trimethylamine N-oxide precursor pathways, such as glycine betaine catabolism (LFCSE = 15.84) and phosphorylcholine phosphatase (LFCSE = 14.22), showed significant positive associations with healthy sleep metrics. Conversely, bacterial functions related to lipopolysaccharide synthesis and export were negatively associated with both cognitive and sleep performance; specifically, heptose II phosphotransferase was inversely related to cognition scores (LFCMoCA = −0.70), while 3-deoxy-D-manno-octulosonic acid kinase demonstrated a negative association with sleep efficiency (LFCSE = −5.71). Additionally, a higher abundance of the lipopolysaccharide exporter was significantly associated with poorer subjective sleep quality (LFCPSQI = 0.17).

Reliability screening of the SE-associated KOs highlighted in Table 5 indicated that the largest coefficients were concentrated among sparsely detected features: malate-CoA ligase (K14067; LFC = 16.04), glycine betaine/dimethylglycine catabolism (K00479/K21833; LFC = 15.84), and phosphorylcholine phosphatase (K21830; LFC = 14.22) each had ≥70% zero abundance across samples and at most 2 non-zero observations within one half of the SE distribution, consistent with near-complete separation. Propionate kinase (K00932; LFC = 9.92) and propionyl-CoA: succinyl-CoA transferase (K22214; LFC = 10.82) showed a similar, though less extreme, sparsity pattern (72.5% zero abundance overall, ≥5 non-zero observations per half). In contrast, the LPS-related KOs (K11211, K16695, K08280) were more consistently detected (55–65% zero abundance, ≥7 non-zero observations per half), and their LFC magnitudes are considered more directly interpretable (Table S2; Figures S4 and S5).

## 4. Discussion

This study examined the independent associations of sleep quality and gut microbiota composition with cognitive function in patients with HF, revealing that poor sleep quality was significantly associated with lower cognitive performance independent of microbiota diversity [49,50]. These findings align with emerging evidence linking sleep disturbances to cognitive decline in HF populations [15,51] and extend current understanding by demonstrating that sleep quality is associated with cognitive function even after accounting for gut microbiota characteristics. The observed association between poor sleep quality and an approximately 3.8-point lower MoCA score may be clinically relevant; however, the clinical significance of this difference in patients with heart failure remains uncertain and should be interpreted cautiously [26,51,52]. Poor sleep quality was significantly associated with lower MoCA scores across all three PSQI-based models, whereas TST and SE were not independently significant in their respective models. Moreover, the models combining PSQI with the Shannon and Simpson indices demonstrated the strongest overall fit, explaining 53–54% of the variance in MoCA scores (adjusted *R*^2^ 0.46–0.47). One possible explanation is that the PSQI captures a broader and multidimensional burden of sleep disturbance, including perceived sleep quality, latency, duration, disruption, and daytime dysfunction, which may be more closely related to cognition than individual sleep dimensions alone.

Alpha diversity and age were consistently independently associated with cognitive function, even after adjustment for sleep parameters, aligning with emerging research implicating the gut microbiota axis in systemic and brain health. Additionally, the associations with microbiota diversity were also dependent on the alpha-diversity metric examined. Shannon and Simpson indices were associated with MoCA scores in several models, whereas Chao1 was not. Because Chao1 primarily estimates microbial richness [43], while Shannon and Simpson indices additionally reflect the distribution, evenness, or dominance of microbial taxa, these findings may suggest that the organization of the microbial community is more relevant to cognitive performance than the number of taxa alone. Although evidence is still limited in HF populations specifically, several human studies have shown significant alterations in gut microbial community structure in HF, with consistent reports of reduced alpha diversity in patients with HF compared to healthy controls. For example, systematic reviews [53] indicate that gut microbial richness and evenness (e.g., Shannon and Chao1 Indices measurements) are significantly lower in HF patients, suggesting a pattern of dysbiosis characterized by loss of beneficial taxa and shifts toward pro-inflammatory species. Furthermore, in our models, coefficients should not be compared directly across diversity indices because the indices operate on different numerical scales. These findings are exploratory and hypothesis-generating and do not demonstrate that any sleep or microbiota characteristic has a unique or causal effect on cognition.

Shannon diversity demonstrated a significant negative association with cognitive performance, whereas Simpson’s dominance index showed a positive association. Although these coefficients appear opposite in direction, they are conceptually concordant because higher Shannon values indicate greater diversity, while higher Simpson *D* values indicate greater dominance and therefore lower diversity [54]. Thus, both findings suggest that greater alpha diversity was associated with lower cognitive performance in this sample. However, these results should not be interpreted as evidence that greater microbial diversity is inherently detrimental, as the biological relevance of diversity depends on the underlying taxonomic and functional composition of the microbial community [54,55]. Indeed, studies in older adults have reported mixed and context-dependent associations between microbial diversity and cognitive outcomes, including both null and significant findings [18,56,57].

The functional metagenomic findings provide mechanistic insights into potential gut–brain-heart axis pathways. The positive associations between SCFA production pathways and improved sleep metrics support the established role of SCFAs as key mediators in microbiota–gut–brain communication [58]. SCFAs, particularly butyrate and propionate, influence brain function through multiple mechanisms including modulation of vagal signaling, regulation of blood–brain barrier integrity, and anti-neuroinflammatory effects [59]. Our finding that SCFA-related pathways correlate with better sleep aligns with evidence that sleep deprivation depletes SCFA-producing bacteria and that SCFA administration can modulate stress responses and emotional processing [60]. The observed associations with TMAO [61] precursor pathways warrant caution, as TMAO exhibits complex, dose-dependent effects on cognitive health, with elevated levels linked to neuroinflammation and accelerated brain aging in some contexts [62] yet showing no clear associations in other population-based studies [63].

Particularly noteworthy are the negative associations between lipopolysaccharide synthesis pathways and both cognitive and sleep outcomes. Lipopolysaccharide, a major component of Gram-negative bacterial cell walls, can translocate across compromised intestinal barriers, a phenomenon exacerbated in HF, and trigger systemic and neuroinflammation via Toll-like receptor 4 (TLR4) activation [64,65]. This gut-derived endotoxemia may represent a critical mechanistic link between intestinal dysbiosis and CI in HF patients, particularly given that HF-associated intestinal congestion and hypoperfusion compromise barrier integrity [56]. The cross-sectional association between lipopolysaccharide export functions and poorer subjective sleep quality is consistent with, but cannot confirm, a potential pathway whereby gut-derived endotoxemia disrupts sleep architecture; prospective studies are needed to determine directionality.

### 4.1. Limitations

Although this study represents an initial effort to apply this research framework in a HF population, several limitations should be considered when interpreting the findings. First, the cross-sectional design (with a limited sample size) precludes any conclusions about directionality or causality among sleep, gut microbiota, and cognitive outcomes; all reported associations are observational. The large number of exploratory and correlated analyses may have increased the risk of false-positive findings, despite FDR correction of the high-dimensional microbiota analyses; therefore, nominally significant associations should be considered hypothesis-generating. Second, residual confounding cannot be excluded because potentially relevant factors, including education, diabetes, chronic kidney disease, depression, antidepressant use, and obstructive sleep apnea, were not included in the adjusted models. The limited sample size restricted our ability to incorporate additional covariates without overfitting; therefore, the observed associations should be interpreted cautiously. The nonsignificant interactions may reflect limited statistical power rather than the absence of true effects. Third, we used subjective measures of sleep quality, which could be affected by existing CI in this population and might not be accurate; therefore, employing objective measures, such as in-home polysomnography or actigraphy, is strongly recommended. Fourth, we attempted to assess overall cognition and memory, along with related attention domains; however, this approach did not provide a comprehensive picture of cognition. Fifth, the exclusive inclusion of patients with heart failure limits the generalizability of the findings to other patient populations. Sixth, several KO-level associations reported in Table 5, particularly for sleep efficiency, exhibited very large log-fold-change estimates (LFC ≈ 14–16) that were traced to low feature prevalence and near-complete separation with respect to the predictor rather than a robust proportional effect; such coefficients from ANCOM-BC2′s log-linear model are better interpreted as presence/absence associations than as proportional fold-changes, and these specific findings should be considered exploratory pending validation in an independent or larger cohort.

### 4.2. Implication for Future Research

Future research should include longitudinal designs to better capture temporal relationships among gut microbiota, sleep parameters, and cognitive trajectories. Repeated microbiota sampling alongside serial cognitive testing would help to determine whether changes in alpha diversity or specific taxa preceded CI or reflect downstream consequences of systemic disease processes. Given the modest sample size in the present study, larger multicenter investigations are warranted to validate the observed associations and interaction effects. Future studies should incorporate multiomics approaches, including metagenomics, metabolomics, and inflammatory biomarkers, to move beyond structural diversity measures and elucidate potential mechanistic pathways (e.g., short-chain fatty acid production, endotoxemia, neuroinflammation). Finally, because sleep architecture appeared to moderate associations between microbiota diversity and cognition, future investigations should integrate objective sleep measurements (e.g., polysomnography or actigraphy) with microbiota profiling to better characterize bidirectional gut, sleep, and brain interactions in HF populations. Finally, we suggest that future studies include a matched healthy cohort, which would enable researchers to compare the diversity and composition of gut bacteria between samples as a first step in analysis.

## 5. Conclusions

Poor sleep quality is independently associated with cognition in HF patients, with effects persisting after adjusting for gut microbiota diversity and clinical covariates. The complex relationships between different alpha diversity indices and cognition, with Shannon diversity showing negative associations and the Simpson index showing positive associations, imply microbial composition and functional capacity may matter more than simple diversity metrics. Functional metagenomic analyses revealed that microbial pathways involved in SCFA production were positively associated with sleep quality, while lipopolysaccharide synthesis and export pathways were negatively associated with both cognitive performance and sleep outcomes. Sleep assessment should be integrated into routine HF management. Therapeutic strategies targeting sleep quality, potentially through microbiota-modulating interventions, may help preserve cognitive function. Longitudinal studies with multi-omics profiling are needed to establish causal pathways and identify specific microbial targets for precision interventions aimed at optimizing both sleep and cognitive health in HF populations.

## Figures and Tables

**Table 1:** Sociodemographic and clinical data of included patients.

Characteristic	*N*	*N* = 59 ^1^
Age	59	73.29 ± 10.80 [37, 93]
Sex	59	
Female		16 (27.11%)
Male		43 (72.89%)
Ethnicity Hispanic/Latina	59	4 (6.8%)
Race	59	
White/Caucasian		51 (86.44%)
Black/African American		6 (10.17%)
Asian		2 (3.39%)
Employment	59	
Full time		2 (3.4%)
Part time		3 (5.1%)
Not employed		7 (12%)
Retired		44 (75%)
Prefer not, declare		3 (5.1%)
Marital status	59	
Single/Never married		11 (18.64%)
Married/living with partner		25 (42.37%)
Divorced/Separated		12 (20.34%)
Widowed		11 (18.64%)
Fruit (per day)	59	1.40 ± 1.66 [0.00, 12.00]
Vegetable (per day)	59	1.38 ± 1.61 [0.00, 10.00]
Total cups of caffeinated beverage (per day)	59	2.60 ± 2.39 [0.00, 12.00]
Total fruit and vegetables (per day)	59	2.21 ± 2.09 [0.00, 11.00]
MoCA	59	23.76 ± 3.76 (12.00, 30.00)
Sleep latency (min)	59	28.45 ± 29.69 [3.00, 180.00]
Prolonged sleep latency (>20 min)	59	28 (45.90%)
Total sleep time (hours)	59	6.98 ± 1.71 [3.50, 12.00]
Short total sleep time (<7 h)	59	26 (42.62%)
Sleep efficiency (%)	59	82.00 ± 0.14.00 [56.00, 100.00]
Poor sleep efficiency (<85%)	59	30 (49.18%)
Poor sleep quality (PSQI > 5)	59	40 (65.57%)
Heart rate (per min)	59	69.08 ± 9.92 [50.00, 96.00]
Systolic blood pressure (mmHg)	59	122.29 ± 12.25 [98.00, 152.00]
Diastolic blood pressure (mmHg)	59	69.49 ± 9.85 [50.00, 88.00]
Level of education	59	
High school or less		26 (44%)
Some college		16 (27%)
Associate degree		4 (6.8%)
Bachelor’s degree		8 (14%)
Graduate degree or professional degree		5 (8.5%)
Drinking behaviors	59	
None		21 (35.5%)
Current		35 (59.3%)
Former		3 (5.2%)
Smoking behaviors	59	
None		28 (47.46%)
Current		5 (8.47%)
Former		26 (44.07%)
HF type (based on EF)	59	
Reduced ejection fraction		36 (61.02%)
Preserved ejection fraction		23 (38.98%)
Left ventricular ejection fraction (%)	59	44.87 ± 10.64 [21.00, 65.00]
**Comorbidities**		
Arrhythmia	59	5 (8.5%)
Arterial fibrillation	59	22 (37%)
Ischemic heart diseases	59	15 (25%)
Hypertension	59	58 (98%)
Stroke	59	1 (1.7%)
Percutaneous transluminal coronary angioplasty	59	43 (73%)
Coronary artery bypass graft	59	15 (25%)
Coronary artery disease	59	36 (61%)
Chronic obstructive pulmonary disease	59	11 (19%)
Chronic kidney disease	59	14 (24%)
Diabetes mellitus	59	27 (46%)
Hyperlipidemia	59	57 (97%)
Hypothyroidism	59	11 (19%)
Gastroesophageal reflux disease	59	10 (17%)
Insomnia	58	3 (5.2%)
Restless leg syndrome	59	1 (1.7%)
Obstructive sleep apnea	59	7 (12%)
Other sleep apnea	59	1 (1.7%)
HIV/AIDS	59	1 (1.7%)
**Medication History**		
Diuretics	59	38 (64%)
Beta blockers	59	48 (81%)
Calcium channel blockers	59	14 (24%)
Angiotensin receptor blockers	59	9 (15%)
Angiotensin-Converting enzyme inhibitors	59	15 (25%)
Entresto (sacubitril/valsartan)	59	20 (34%)
Antiplatelet	59	50 (85%)
DPP-4 inhibitors	59	6 (10%)
Statins	59	38 (64%)
Selective serotonin reuptake inhibitors	59	6 (10%)
Serotonin-Norepinephrine reuptake inhibitor	59	3 (5.1%)
Tricyclic antidepressants	59	2 (3.4%)
Benzodiazepines	59	2 (3.4%)
Antipsychotics	59	1 (1.7%)
Antiarrhythmics	59	9 (15%)
GLP-1 agonist	59	6 (10%)
How many days, get samples	40	13.50 ± 11.57 [1.00, 43.00]
Fecal Sample reception	61	
No		18 (30%)
Yes		40 (66%)
Refused		3 (4.9%)
**Microbiota**		
Simpson	40	0.05 ± 0.02
Chao1	40	246.00 ± 72.81
Observed (ASVs)	40	241.20 ± 71.28
Shannon	40	3.58 ± 0.39

^1^ n (%); Mean ± SD [Min, Max].

**Table 2:** Linear regression results of beta diversity and MoCA.

	β	SE	t	*p* Value
Intercept	41.87	4.51	9.28	<0.001
Poor Sleep Quality (vs. good)	−3.76	1.27	−2.95	0.006
Axis.1	−3.18	3.23	−0.98	0.332
Axis.2	−1.92	3.63	−0.53	0.600
Age	−0.23	0.05	−4.20	<0.001
Sex	−1.64	1.42	−1.16	0.255
LVEF	0.05	0.05	0.95	0.350

**Table 4:** Summary of multiple regression models of MoCA by sleep quality and microbiota diversity.

Predictor	Shannon Model (PSQI)	Chao1 Model (PSQI)	Simpson Model (PSQI)	Shannon Model (TST)	Chao1 Model (TST)	Simpson Model (TST)	Shannon Model (SE)	Chao1 Model (SE)	Simpson Model (SE)
Intercept	50.03 (5.43) ***	41.74 (4.30) ***	35.95 (4.47) ***	47.10 (6.17) ***	36.04 (5.00) ***	30.31 (4.73) ***	49.19 (6.04) ***	38.05 (4.85) ***	31.42 (4.86) ***
Sleep Metric ^a^	−3.31 (1.15) **	−3.88 (1.18) **	−3.22 (1.15) **	1.34 (1.12)	1.21 (1.25)	1.03 (1.13)	0.21 (1.30)	−0.39 (1.39)	0.02 (1.26)
Microbiota Index ^b^	−3.45 (1.39) *	−0.01 (0.01)	57.43 (21.71) *	−4.27 (1.48) **	−0.01 (0.01)	69.47 (23.31) **	−4.45 (1.57) **	−0.01 (0.01)	72.71 (23.98) **
Age	−0.18 (0.05) ***	−0.19 (0.05) **	−0.20 (0.05) ***	−0.15 (0.06) **	−0.17 (0.06) *	−0.18 (0.05) **	−0.17 (0.06) **	−0.18 (0.06) **	−0.19 (0.05) **
Sex (Male)	−0.84 (1.36)	−1.48 (1.39)	−0.88 (1.33)	0.76 (1.34)	0.19 (1.45)	0.65 (1.32)	0.96 (1.60)	0.04 (1.70)	0.74 (1.55)
LVEF	0.06 (0.05)	0.06 (0.05)	0.05 (0.05)	0.05 (0.05)	0.04 (0.06)	0.04 (0.05)	0.05 (0.05)	0.05 (0.06)	0.04 (0.05)
*R* ^2^	0.53	0.48	0.54	0.44	0.33	0.44	0.41	0.31	0.43
Adj. *R*^2^	0.46	0.40	0.47	0.35	0.23	0.36	0.33	0.21	0.35

Note. Table entries are unstandardized coefficients (B) with standard errors (SE) in parentheses. ^a^ Sleep metrics include Poor sleep quality (first 3 models), Short sleep time (middle 3 models), and Poor efficiency (final 3 models). ^b^ Microbiota indices correspond to the header (Shannon diversity, Chao1 index, or Simpson index) for that specific model. * *p* < 0.05, ** *p* < 0.01, *** *p* < 0.001.

**Table 5:** Associations between specific gut microbiota metabolic pathways and measures of cognition and sleep quality.

Theme	Bacterial Ortholog (KO)	Relevant Variable(s)	Direction and Log-Fold Change (LFC)	Interpretation
Short-Chain Fatty Acids (SCFAs)	Propionate kinase (K00932)	Sleep (PSQI, TST, SE)	Positive: PSQI (−0.24 ^a^), TST (0.77), SE (9.92)	Better sleep quality, duration, and efficiency are all associated with an enrichment of propionate-producing bacteria.
	Propionyl-CoA:succinyl-CoA transferase (K22214)	Sleep (PSQI, TST, SE)	Positive: PSQI (−0.25 ^a^), TST (0.84), SE (10.81)	Enrichment of these enzymes suggests that efficient sleepers have a microbiota more capable of generating beneficial SCFAs.
	Malate-CoA ligase (K14067) ^b^	Sleep (PSQI, TST, SE)	Positive: PSQI (−0.52 ^a^), TST (2.23), SE (16.03)	This core SCFA metabolic pathway shows a massive positive correlation with sleep efficiency (SE).
TMAO Precursors/Choline	Glycine betaine/Dimethylglycine catabolism (K00479/K21833)	Sleep (PSQI, TST, SE)	Positive: PSQI (−0.60 ^a^), TST (2.52), SE (15.83)	Better sleep is associated with bacteria that process precursors used in the eventual production of TMAO.
	Phosphorylcholine phosphatase (K21830)	Sleep (PSQI, TST, SE)	Positive: PSQI (−0.36 ^a^), TST (2.32), SE (14.22)	Choline processing capabilities are highly enriched in individuals with high sleep duration and efficiency.
Lipopolysaccharides (LPS)/Endotoxins	Heptose II phosphotransferase (K02850)	Cognition (MoCA)	Negative: MoCA (−0.70)	Higher cognitive performance is associated with a lower abundance of bacteria involved in synthesizing this core LPS component.
	3-deoxy-D-manno-octulosonic acid (KDO) kinase (K11211)	Sleep (TST, SE)	Negative: TST (−0.39), SE (−5.70)	Better sleep duration and efficiency are associated with reduced levels of this critical endotoxin/LPS linker.
	LPS exporter/O-acetyltransferase (K16695/K08280)	Sleep (PSQI)	Negative: PSQI (0.16, 0.33)	Poorer subjective sleep (high PSQI scores) is positively correlated with bacterial ability to modify and export LPS.

^a^ *Note: For the PSQI variable, a negative LFC indicates higher abundance in individuals with lower PSQI scores (better sleep).* ^b^ *Malate-CoA ligase (K14067), glycine betaine/dimethylglycine catabolism (K00479/K21833), and phosphorylcholine phosphatase (K21830) had ≥70% zero abundance across samples and ≤2 non-zero observations within one half of the sleep-efficiency distribution; these LFC estimates are likely inflated by near-complete separation and should be interpreted as presence/absence associations rather than proportional fold-changes (see Discussion, Limitations; full diagnostic in* Supplementary Table S2*).*

## Data Availability

Data from this study are available to researchers upon reasonable request to the corresponding author. This article is part of Hesam Varpaei’s doctoral dissertation to fulfill the requirements of the PhD in Nursing at Michigan State University.

## Supplementary Materials

The following supporting information can be downloaded at https://www.mdpi.com/article/10.3390/medicina62091643/s1. Supplementary File S2: Analysis of Compositions of Microbiomes with Bias Correction (version 2) Differential Abundance Analysis of Kyoto Encyclopedia of Genes and Genomes Orthologs Associated with Sleep and Cognitive Measures; Figures S1: Proportional Distribution of Mean Relative Abundance (Top 5) at the Phylum Level; Figure S2: Proportional Distribution of Mean Relative Abundance (Top 10) at the Genus Level; Figure S3: Distribution of PICTUSt2 NSTI values across Amplicon Sequence Variant, Figure S4: Sleep efficiency KO log-fold change (LCF) reliability, Figure S5: ANCOM-BC2 log-fold change (LCF), per 1.0-unit increase in sleep efficiency; Table S1: Nearest Sequenced Taxon Index (NSTI) summary table, Table S2: Log-fold change (LCF) reliability per 1.0 SE and per 0.10 SE, Table S3: Cognitive Impairment by Sleep Groups; Table S4: Alpha Diversity by Cognitive and Sleep Groups, Table S5: Moderation Analysis—Alpha Diversity × Sleep Variable Interaction on MoCA, Table S6: Montreal Cognitive Assessment (MoCA) (Continuous), Table S7: PSQI Global Score (Continuous), Table S8: Total Sleep Time (TST) (Continuous), Table S9: Sleep Latency (Continuous), Table S10: Sleep Efficiency (Continuous). Reference [66] is cited in the Supplementary Materials.
