# Supplementary material for: Exploring the Relationship Between Sleep Disturbances, Gut Microbiota, and Cognition in Patients with Heart Failure: A Cross-Sectional Study
Source: Medicina (Kaunas). 2026 Aug 27;62(9):1643. doi: 10.3390/medicina62091643 (PMC13608842; doi:10.3390/medicina62091643)
Supplement: Supplementary file 1 [file medicina-62-01643-s001.zip › Supplements S1_REV2 final.pdf]

Supplements

Sample Size and Power

Initial sample size calculations based on the detection of a small-to-moderate association (Cohen's $f^2 = 0.10$) using multiple linear regression with two predictors with $\alpha = 0.05$ and beta= 0.20, estimated a sample size of 100 participants. To account for anticipated attrition and unusable fecal specimens, we planned to recruit 115 participants. In practice, recruitment yield and specimen's usability were lower than anticipated, and the final analytic sample included 59 participants (Figure 1), with 40 providing usable fecal samples. In addition, microbiome sequencing and processing costs limited the number of samples that could be analyzed within the available budget. Accordingly, analyses involving microbiome measures were conducted as exploration and focused on estimation (effect sizes with 95% confidence intervals) rather than definitive hypothesis testing. Also, no post-hoc power analysis was conducted, as it does not provide useful information for interpreting results (Heinsberg & Weeks, 2022).

Heinsberg, L. W., & Weeks, D. E. (2022). Post hoc power is not informative. *Genetic Epidemiology*, *46*(7), 390–394. https://doi.org/10.1002/gepi.22464

Figure S1

Proportional Distribution of Mean Relative Abundance (Top 5) at the Phylum Level

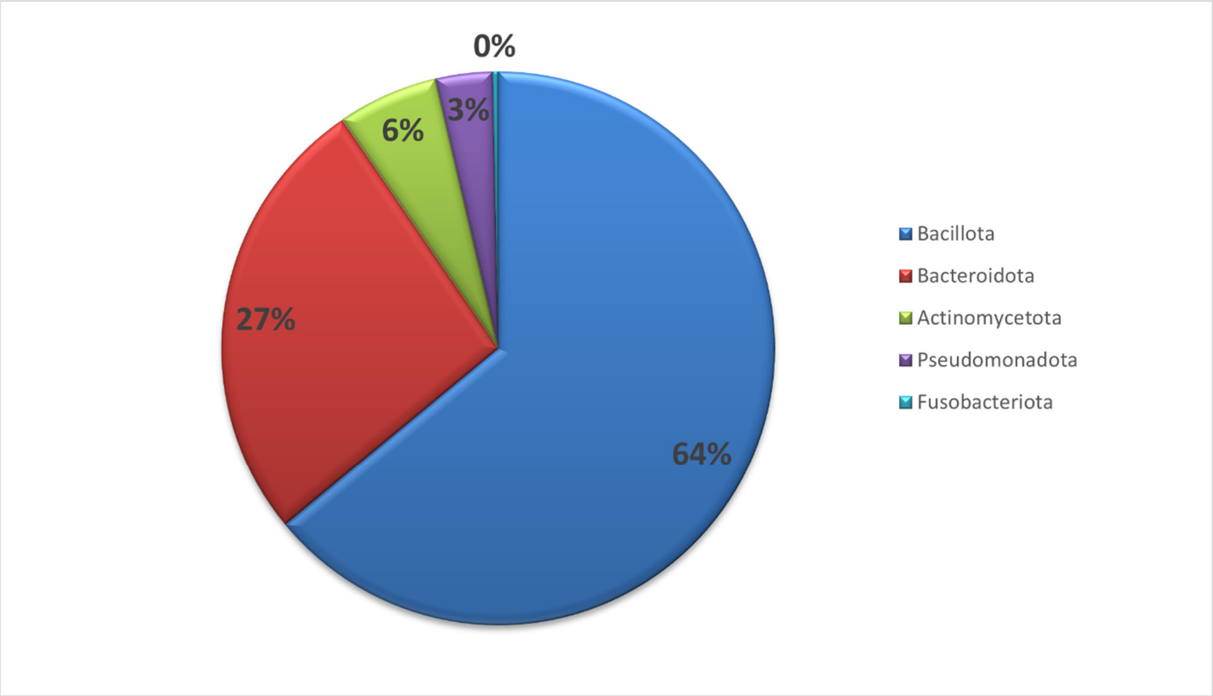

Figure S2

Proportional Distribution of Mean Relative Abundance (Top 10) at the Genus Level

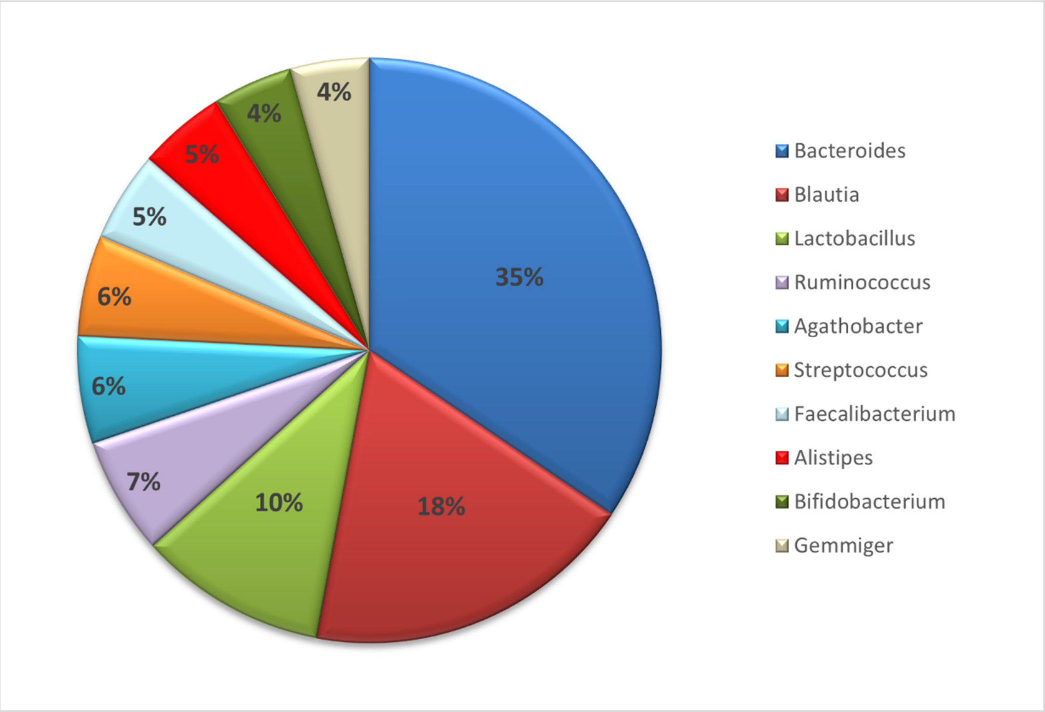

Figure S3

Distribution of PICTUSt2 NSTI values across Amplicon Sequence Variant

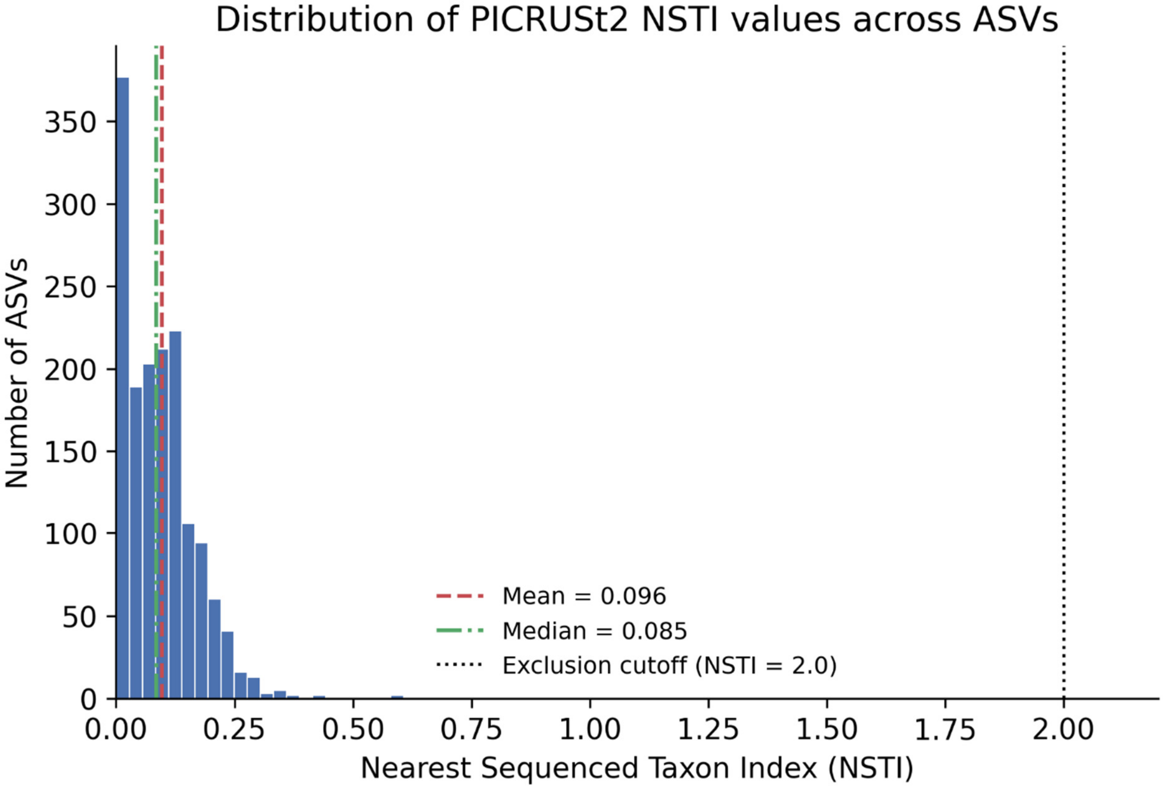

Figure S4

Sleep efficiency KO log-fold change (LCF) reliability

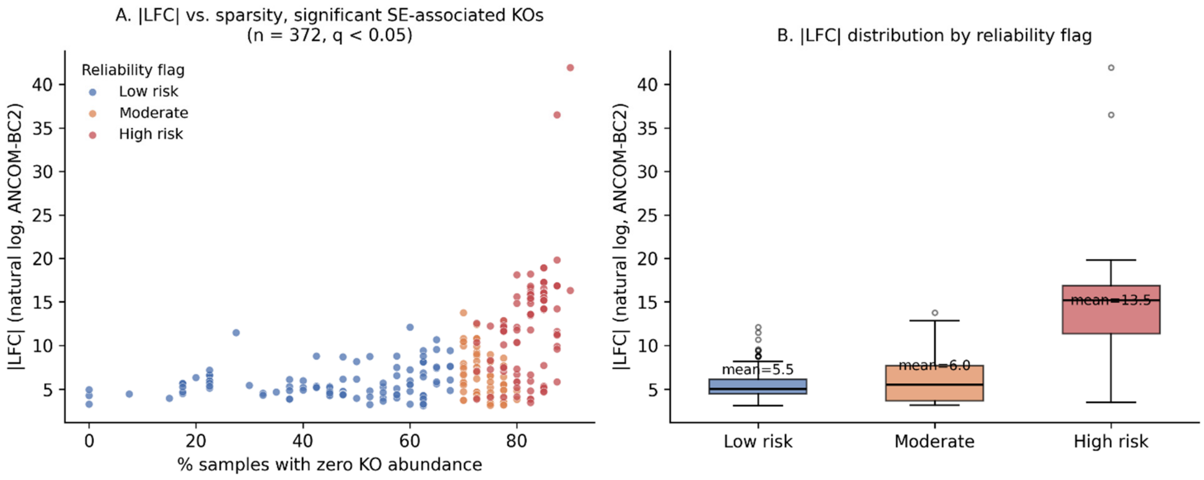

Figure S5

ANCOM-BC2 log-fold change (LCF), per 1.0-unit increase in sleep efficiency

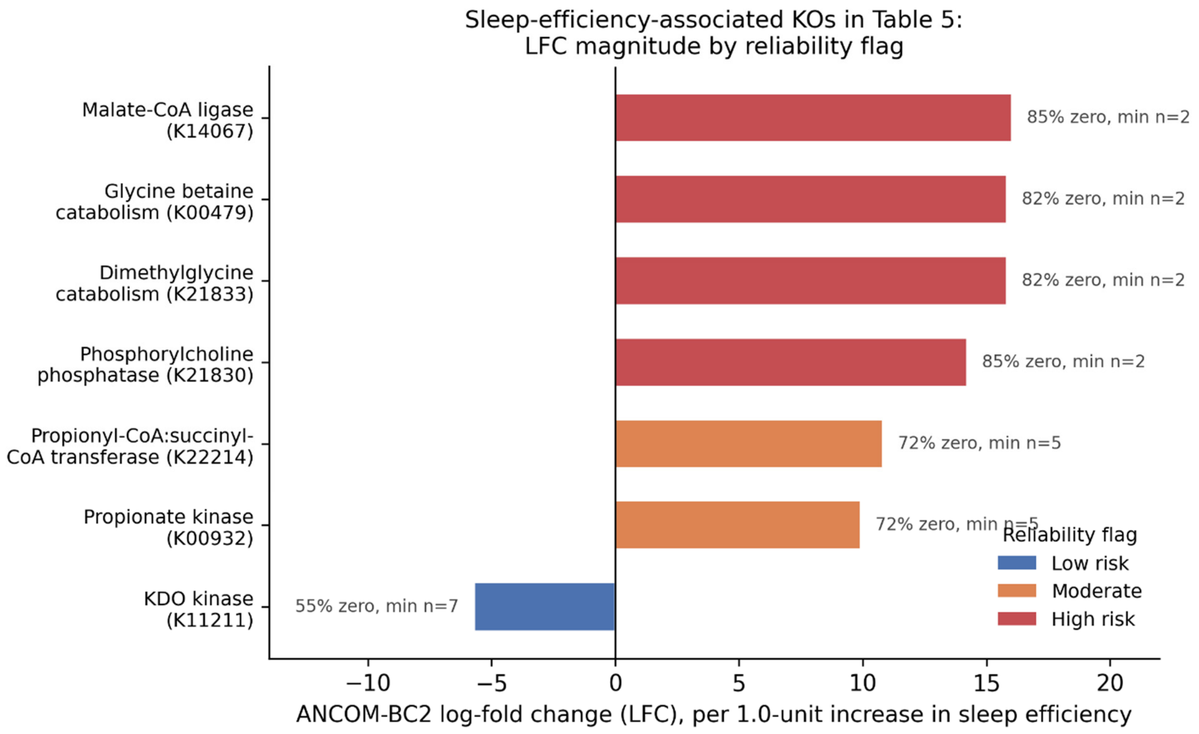

Table S1. Nearest Sequenced Taxon Index (NSTI) summary table

Metric	Value
Number of ASVs (bacterial)	1557
Mean NSTI	0.0962
SD	0.096
Median NSTI	0.0847
IQR (25th-75th percentile)	0.0289 - 0.1339
Min NSTI	0.00015
Max NSTI	1.1048
ASVs excluded (NSTI > 2.0, default max NSTI cutoff)	0 (0.0%)
% ASVs with NSTI <= 0.15	80.50%
% ASVs with NSTI <= 0.2	91.10%

Table S2. Log-fold change (LCF) reliability per 1.0 SE and per 0.10 SE

KO	Description	Pathway theme	LFC (per 1.0 SE)	LFC (per 0.10 SE)	Q value	% zero abundance (n=40)	Non-zero, low-SE half (n=20)	Non-zero, high-SE half (n=20)	Min non-zero (either half)	Reliability flag
K14067	Malate-CoA ligase	SCFA	16.036	1.604	4.284E-03	85.0	2	4	2	High risk
K21833	Dimethylglycine catabolism	TMAO precursor	15.838	1.584	2.679E-03	82.5	2	5	2	High risk
K00479	Glycine betaine catabolism	TMAO precursor	15.838	1.584	2.679E-03	82.5	2	5	2	High risk
K21830	Phosphorylcholine phosphatase	TMAO precursor	14.223	1.422	9.502E-03	85.0	2	4	2	High risk
K22214	Propionyl-CoA:succinyl-CoA transferase	SCFA	10.817	1.082	3.731E-03	72.5	5	6	5	Moderate
K00932	Propionate kinase	SCFA	9.924	0.992	3.731E-03	72.5	5	6	5	Moderate
K11211	3-deoxy-D-manno-octulosonic acid (KDO) kinase	LPS/Endotoxin	-5.709	-0.571	1.428E-03	55.0	11	7	7	Low risk

Scope: The 7 KOs from manuscript Table 5 with a reported sleep-efficiency (SE)-associated log-fold change (LFC). Heptose II phosphotransferase (K02850, MoCA outcome) and the LPS exporter/O-acetyltransferase KOs (K16695/K08280, PSQI outcome) are excluded as they have no SE-associated LFC.
LFC (per 1.0 SE): ANCOM-BC2 log-fold change (natural log) per 1.0-unit (100-percentage-point) increase in sleep efficiency, as originally reported.
LFC (per 0.10 SE): Same estimate rescaled to a realistic 10-percentage-point increase in SE, for interpretability.
% zero abundance (n=40): Percentage of the 40 study samples in which this KO had zero predicted abundance.
Non-zero, low/high-SE half: Number of samples with non-zero abundance among the 20 lowest- and 20 highest-SE participants (split at median SE), respectively.
Min non-zero (either half): Smaller of the two non-zero counts; low values indicate near-complete separation with respect to SE.
Reliability flag: High risk: >=70% zero overall AND <=3 non-zero samples on one side of the SE split (LFC likely inflated by near-separation, interpret as presence/absence signal). Moderate: >=70% zero overall but better-populated on both sides. Low risk: <70% zero overall (LFC magnitude adequately supported by the data).

Table S3. Cognitive Impairment by Sleep Groups

**Variable**	**Group**	**N**	**CI <26, n (%)**	**Normal ≥26, n (%)**
**Overall**		59	40 (67.8%)	19 (32.2%)
**PSQI**	Good sleep (=<5)	19	11 (57.9%)	8 (42.1%)
	Poor sleep	40	29 (72.5%)	11 (27.5%)
**Sleep Duration**	Adequate	33	25 (75.8%)	8 (24.2%)
	Short	26	15 (57.7%)	11 (42.3%)
**Sleep Efficiency**	Good	29	20 (69.0%)	9 (31.0%)
	Poor (<85%)	30	20 (66.7%)	10 (33.3%)

Table S4. Alpha Diversity by Cognitive and Sleep Groups (Mean ± SD)

Metric	Group	N	Mean ± SD	Median [IQR]	p-value*
Shannon					
MoCA	Normal ≥26	14	3.38 ± 0.44	3.40 [3.05–3.59]	0.008
	CI <26	26	3.70 ± 0.32	3.67 [3.55–3.90]	
PSQI	Good sleep (=<5)	14	3.54 ± 0.44	3.59 [3.37–3.82]	0.528
	Poor sleep	26	3.61 ± 0.37	3.62 [3.50–3.84]	
TST	Adequate	23	3.63 ± 0.44	3.65 [3.49–3.89]	0.290
	Short	17	3.53 ± 0.33	3.53 [3.35–3.73]	
SE	Good	22	3.55 ± 0.44	3.54 [3.37–3.82]	0.325
	Poor (<85%)	18	3.63 ± 0.33	3.65 [3.54–3.84]	
Chao1					
MoCA	Normal ≥26	14	215.6 ± 76.9	207.0 [177.0–239.7]	0.048
	CI <26	26	262.3 ± 66.3	263.1 [227.7–295.3]	
PSQI	Good sleep (=<5)	14	251.8 ± 92.0	255.5 [195.4–302.9]	0.747
	Poor sleep	26	242.8 ± 62.0	248.0 [202.6–277.8]	
TST	Adequate (>7h)	23	262.8 ± 82.3	278.0 [204.4–317.1]	0.075
	Short	17	223.2 ± 51.5	241.8 [197.5–250.2]	
SE	Good	22	239.7 ± 82.5	240.8 [175.5–302.9]	0.677
	Poor (<85%)	18	253.6 ± 60.4	248.1 [215.9–277.8]	
Simpson					
MoCA	Normal ≥26	14	0.070 ± 0.032	0.061 [0.055–0.086]	0.012
	CI <26	26	0.050 ± 0.014	0.051 [0.039–0.055]	
PSQI	Good sleep (=<5)	14	0.061 ± 0.032	0.056 [0.041–0.062]	0.747
	Poor sleep	26	0.055 ± 0.019	0.052 [0.042–0.061]	
TST	Adequate	23	0.053 ± 0.022	0.051 [0.037–0.059]	0.173

	Short	17	0.061 ± 0.026	0.054 [0.048–0.063]	
SE	Good	22	0.059 ± 0.028	0.055 [0.041–0.063]	0.563
	Poor (<85%)	18	0.054 ± 0.019	0.049 [0.042–0.059]	
Observed					
MoCA	Normal ≥26	14	212.5 ± 75.6	201.5 [176.3–237.3]	0.050
	CI <26	26	256.7 ± 65.1	256.5 [220.3–291.5]	
PSQI	Good sleep	14	246.4 ± 89.3	250.0 [188.3–297.3]	0.755
	Poor sleep	26	238.4 ± 61.3	243.5 [199.3–271.5]	
TST	Adequate	23	257.3 ± 80.7	273.0 [203.5–312.0]	0.085
	Short	17	219.4 ± 50.6	239.0 [195.0–249.0]	
SE	Good	22	234.5 ± 80.7	231.0 [174.0–297.3]	0.596
	Poor (<85%)	18	249.3 ± 59.0	244.5 [212.0–271.5]	

*Wilcoxon signed-rank test

Table S5. Moderation Analysis — Alpha Diversity × Sleep Variable Interaction on MoCA

Diversity Metric	Sleep Moderator	N	R^2	Adj. R^2	Interaction p-value
Observed	SE (binary)	40	0.342	0.223	0.215
Chao1	SE (binary)	40	0.343	0.223	0.228
Simpson	PSQI category	40	0.554	0.473	0.276
Shannon	TST (binary)	40	0.446	0.346	0.470
Simpson	SE (binary)	40	0.434	0.332	0.625
Simpson	TST (binary)	40	0.447	0.347	0.667
Chao1	PSQI category	40	0.479	0.384	0.774
Observed	PSQI category	40	0.476	0.381	0.792
Shannon	PSQI category	40	0.529	0.443	0.838
Chao1	TST (binary)	40	0.330	0.208	0.841
Observed	TST (binary)	40	0.328	0.206	0.899
Shannon	SE (binary)	40	0.414	0.308	0.972

Table S6 – Montreal Cognitive Assessment (MoCA) (Continuous)

Taxa Level	Taxon	exp(LFC)	SE	W	p_value	q_value
Phylum	Fusobacteriota	**0.4173**	0.079	-11.055	0.000004	0.00004
Genus	Fusobacterium	**0.5294**	0.075	-8.488	0.00003	0.00694
Genus	Slackia	**0.5363**	0.076	-8.166	0.00018	0.04412
Species	*P. grossensis*	**2.3187**	0.079	10.693	0.00175	0.62950
Species	CAG-352	**1.8368**	0.085	7.157	0.00202	0.72430

Table S7 – PSQI Global Score (Continuous)

Taxa Level	Taxon	exp(LFC)	SE	W	p_value	q_value
Phylum	Fusobacteriota	**0.6907**	0.045	-8.308	0.00003	0.00033
Genus	Porphyromonas	**0.7603**	0.035	-7.923	0.00010	0.02384
Genus	Tyzzerella	**0.4369**	0.032	-25.920	0.00013	0.03086
Genus	Peptococcus	**0.8057**	0.037	-5.869	0.00016	0.03845
Species	*V. dispar*	**1.2892**	0.033	7.757	0.00011	0.04016
Species	Peptococcus	**0.7929**	0.036	-6.397	0.00013	0.04537
Species	Tyzzerella	**0.4431**	0.031	-25.909	0.00013	0.04540

Table S8 – Total Sleep Time (TST) (Continuous)

**Taxa Level**	**Taxon**	**exp(LFC)**	**SE**	**W**	**p_value**	**q_value**
Phylum	Fusobacteriota	**2.0627**	0.144	5.018	0.00103	0.01029
Genus	Pseudomonas	**8.7670**	0.135	16.093	0.00052	0.12840
Species	*C. stercoris*	**2.4303**	0.134	6.633	0.00006	0.02112

Table S9 – Sleep Latency (Continuous)

**Taxa Level**	**Taxon**	**exp(LFC)**	**SE**	**W**	**p_value**	**q_value**
Phylum	Campylobacterota	**0.9666**	0.006	-5.789	0.00443	0.04426
Genus	Mogibacterium	**1.0747**	0.007	11.004	0.0000001	0.00001
Genus	Bacteroidia Class	**0.9213**	0.006	-13.638	0.0000003	0.00006
Genus	Shuttleworthia	**1.2214**	0.004	45.965	0.000001	0.00032
Species	Bacteroidia Class	**0.9231**	0.006	-13.477	0.0000003	0.00010
Species	Shuttleworthia	**1.2153**	0.004	46.007	0.000001	0.00048

Table S10 – Sleep Efficiency (Continuous)

Taxa Level	Taxon	exp(LFC)	SE	W	p_value	q_value
Phylum	Synergistota	**0.8816**	0.025	-5.126	0.01438	0.14380
Genus	Segatella	**0.8940**	0.023	-4.892	0.00273	0.66680
Species	Segatella	**0.8940**	0.023	-4.796	0.00301	0.99999
